# Supplementary material for: EnzML: multi-label prediction of enzyme classes using InterPro signatures
Source: BMC Bioinformatics. 2012 Apr 25;13:61. doi: 10.1186/1471-2105-13-61 (PMC3483700; doi:10.1186/1471-2105-13-61)
Supplement: Addtional file 5 — The Java code to format the data files, evaluate and predict. The file enzml_java_code.tar.gz contains the Java code used to format database data to ARFF and XML formats, to execute cross and train-test (jackknife) evaluations and to record evaluation results to database. More information is included in the readme.txt file and the Javadoc files. The code can be used with a MySQL database. To use a different database software, other JDBC drivers might be required. [file 1471-2105-13-61-S5.gz › java_code/utils/doc/cern/jet/random/class-use/Pareto.html]

Uses of Class cern.jet.random.Pareto


---


|  |  |  |
| --- | --- | --- |
| **Overview** \| **Package** \| **Class** \| **Use** \| **Tree** \| **Deprecated** \| **Index** \| **Help** | |  |
| PREV   NEXT | **FRAMES**    **NO FRAMES**     **All Classes** |  |


---


## **Uses of Class cern.jet.random.Pareto**

| Packages that use Pareto | |
| --- | --- |
| **uk.ac.ed.inf.utils.stats** |  |

Uses of Pareto in uk.ac.ed.inf.utils.stats

| Subclasses of Pareto in uk.ac.ed.inf.utils.stats | |
| --- | --- |
| `class` | `PseudoTruncatedPareto`             A truncated pareto distribution which stores a big lists of power-law extracted random numbers. |
| `class` | `TruncatedPareto`             A utility class to extract from a truncated Pareto (power-law, long-tail) distribution. |

| Methods in uk.ac.ed.inf.utils.stats that return Pareto | |
| --- | --- |
| `Pareto` | `TruncatedPareto.getPareto()` |

| Methods in uk.ac.ed.inf.utils.stats with parameters of type Pareto | |
| --- | --- |
| `static java.util.TreeMap<java.lang.Integer,java.lang.Integer>` | `StatUtils.paretoFrequencies(Pareto pareto, int numberOfExtractions)`             Extracts numbers from a pareto distribution for the given number of times and records the freqencies with which the integers have been extracted |

---


|  |  |  |
| --- | --- | --- |
| **Overview** \| **Package** \| **Class** \| **Use** \| **Tree** \| **Deprecated** \| **Index** \| **Help** | |  |
| PREV   NEXT | **FRAMES**    **NO FRAMES**     **All Classes** |  |


---
